# Electroencephalography Functional Network Responses to Immersive Virtual Reality in Alzheimer Disease and Mild Cognitive Impairment: Exploratory Single-Session 3-Group Repeated-Measures Study

**DOI:** 10.2196/95160

**Published:** 2026-09-04

**Authors:** Yi Hao Yao, SHIBO XU, Eun-Seong Kim, Eun-Ah Kim, Yan-Xiong Wang, Hyun Soo Kim, Do Hoon Kim, Jun-Ge Liang, Young-Kee Shin, Nam-Young Kim

**Affiliations:** 1 RF Bio Center Department of Electronic Engineering Kwangwoon University Seoul, Seoul Republic of Korea; 2 Department of Molecular Medicine and Biopharmaceutical Sciences Laboratory of Molecular Pathology and Cancer Genomics Seoul National University Seoul, Seoul Republic of Korea; 3 Kimsbiolab Co., Ltd. Seoul Republic of Korea; 4 Department of Electronic Engineering Kwangwoon University Seoul, Seoul Republic of Korea; 5 Chuncheon Sacred Heart Hospital and Mind-Neuromodulation Laboratory Department of Psychiatry Hallym University Seoul, Gangwon-do Republic of Korea; 6 Molecular & Cellular Reprogramming Center School of Advanced Biotechnology, College of Institute of Science and Technology Konkuk University Seoul, Seoul Republic of Korea

**Keywords:** AD, Alzheimer disease, EEG, electroencephalography, functional connectivity, graph theory, mild cognitive impairment, virtual reality

## Abstract

**Background:**

Alzheimer disease (AD) is increasingly conceptualized as a disorder of large-scale brain network disruption rather than isolated regional dysfunction. Immersive virtual reality (VR) may provide a controlled sensory challenge for neurophysiological assessment, but its association with whole-brain electroencephalography (EEG) functional network organization across AD and mild cognitive impairment (MCI) remains insufficiently characterized.

**Objective:**

This study examined whether immersive VR exposure was associated with state-dependent changes in EEG-derived functional network organization among older adults with AD, MCI, and normal cognition (NC).

**Methods:**

This exploratory single-session, 3-group repeated-measures EEG study included 60 older adults: 20 with AD, 20 with MCI, and 20 participants with NC. During a single visit, participants underwent repeated within-session EEG recordings during pre-VR resting, immersive VR exposure, and post-VR resting states. VR was delivered through a head-mounted display presenting a passive first-person roller-coaster environment. Functional connectivity was quantified using phase-locking value (PLV) across multiple frequency bands, and global efficiency (GE), modularity, and nodal strength were calculated to characterize network topology. Between-group differences within each state were assessed using 1-way ANOVA with post hoc pairwise comparisons, whereas within-group state changes were evaluated using paired-sample 2-tailed *t* tests. Benjamini-Hochberg false discovery rate (FDR) correction was applied to inferential graph-theoretical analyses. PLV matrices, nodal strength maps, and connectivity graphs were interpreted descriptively.

**Results:**

In the broadband range, GE was higher in the AD group than in the NC group during the pre-VR resting state (raw *P*=.009; FDR-adjusted *P*=.04), whereas the AD-NC difference was not statistically significant during VR exposure or the post-VR resting state. Baseline modularity was also higher in AD than in NC and remained significant after FDR correction; no significant between-group modularity differences were detected during VR exposure. Within-group analyses showed an FDR-corrected increase in GE from pre-VR to VR in NC participants and from pre-VR to post-VR in participants with MCI. No within-group GE change in AD remained significant after FDR correction. Descriptive PLV, nodal strength, and connection maps suggested state-associated changes in hemispheric distribution and high-frequency connectivity patterns. However, high-frequency GE findings did not remain significant after FDR correction and were therefore interpreted as exploratory.

**Conclusions:**

In this exploratory single-session, 3-group repeated-measures EEG study, immersive VR exposure was associated with short-term, state-dependent changes in PLV-derived EEG functional network organization among older adults with AD, MCI, and NC. Graph-theoretical analysis demonstrated the feasibility of using repeated within-session VR-EEG measurements to characterize acute network responsiveness across cognitive groups. However, descriptive connectivity maps were not treated as confirmatory evidence, and the absence of a non-VR comparison condition and concurrent cognitive or clinical outcomes precludes conclusions regarding causality or therapeutic efficacy. Larger controlled and longitudinal studies are needed to establish the reproducibility and clinical relevance of these findings.

## Introduction

Alzheimer disease (AD) is an insidious and progressive neurodegenerative disorder and the leading cause of dementia [1-3]. It is clinically characterized by progressive cortical atrophy, cognitive decline, and behavioral dysfunction [4,5]. More than 60 million people worldwide currently live with AD, and projections suggest this number will approach 80 million by 2030. AD remains incurable and predominantly affects older adults, with an average life expectancy of only 4 to 8 years after diagnosis [6]. Pharmacological therapies remain the standard approach for slowing disease progression, yet supportive care alone often fails to preserve quality of life [5,7]. Identification of effective nonpharmacological interventions therefore remains essential. Virtual reality (VR) technology has advanced substantially, and immersive neural stimulation may exert distinct modulatory effects on functional brain connectivity [8,9]. This study aims to characterize brain network differences among AD, mild cognitive impairment (MCI), and normal cognition (NC) groups and to investigate whether VR stimulation is associated with alterations in functional brain network organization [10,11]. Electroencephalography (EEG), which records cortical oscillations using scalp electrodes with high temporal resolution, provides a robust tool for this investigation [12-16].

Previous studies have identified hallmark network abnormalities in AD and MCI, including altered phase synchrony, reduced global efficiency (GE), disrupted modular organization, and interhemispheric connectivity imbalance [17,18]. Phase-based metrics such as phase-locking value (PLV) show reduced synchronization in AD, especially across long-range connections, indicating impaired large-scale coordination. GE measures show more complex patterns. Healthy aging typically correlates with reduced efficiency, whereas AD may show paradoxically increased resting-state GE, which may reflect pathological overintegration or loss of modular constraints rather than enhanced processing. Modularity analyses further indicate that AD networks become excessively segregated, with rigid community boundaries that restrict flexible reconfiguration [1,19,20].

MCI represents a transitional stage between healthy aging and dementia and is characterized by heterogeneous and often delayed network responses. Evidence shows that MCI exhibits network properties that are intermediate between those of NC and AD groups, but with significantly reduced dynamic adaptability [21,22]. Most existing EEG studies rely on resting-state recordings, which limit understanding of how pathological networks respond to external stimulation or cognitive engagement.

VR represents a promising nonpharmacological approach for modulating brain function in older adults and neurodegenerative populations. Through immersive, multisensory input, VR can simultaneously engage distributed perceptual, attentional, and sensorimotor networks. Neuroimaging studies suggest that VR stimulation may influence neuroplastic processes, alter hemispheric activity patterns, and promote functional reorganization [23-25]. However, the mechanisms through which VR dynamically modulates large-scale EEG networks in AD and MCI, particularly across frequency bands and temporal stages, remain poorly defined [26,27].

A key unresolved question is whether immersive VR exposure is associated with measurable changes in EEG functional network organization in AD and MCI and whether the observed within-session patterns differ descriptively from those in cognitively normal older adults. To address this question, we conducted an exploratory, single-session, 3-group repeated-measures EEG study using pre-VR resting, immersive VR exposure, and post-VR resting states. By integrating multiband PLV-derived connectivity with graph-theoretical metrics, we aimed to characterize group-related network organization, examine state-associated network changes, and evaluate the feasibility of VR-EEG as an experimental framework for assessing acute neurophysiological responsiveness [28-30]. We hypothesized that immersive VR exposure would be associated with state-dependent changes in EEG-derived functional networks and that the 3 diagnostic groups would show distinct within-session response patterns.

## Methods

### Ethical Considerations

This study was conducted in accordance with the Declaration of Helsinki and was approved by the Institutional Review Board (IRB) of Kwangwoon University, Republic of Korea, under IRB number 7001546-202300614-HR(SB)-005-01. Written informed consent was obtained from all participants before enrollment.

Participant confidentiality was strictly protected throughout the study. All EEG recordings and demographic information were anonymized and deidentified before analysis. No personally identifiable information was included in the study database, and no identifiable participant images are presented in this manuscript.

Participants were recruited on a voluntary basis and did not receive financial compensation for participation.

### Study Design and Setting

This was an exploratory, nonrandomized, single-session, 3-group repeated-measures EEG study. Participants with AD, MCI, and NC underwent repeated EEG measurements during pre-VR resting, immersive VR exposure, and post-VR resting states within 1 experimental session. Diagnostic group constituted the between-participant factor, and experimental state constituted the within-participant repeated-measures factor. All participants underwent the same VR procedure. No randomization, treatment allocation, separate non-VR comparison condition, or longitudinal follow-up was included. The study was designed to characterize short-term EEG functional network responses rather than to evaluate therapeutic efficacy.

### Sampling Procedures

Participants were recruited through advertisements at Chuncheon Sacred Heart Hospital within 1 month before the experimental sessions. Patients with AD and individuals with MCI were recruited from hospital patients whose diagnoses and clinical status were confirmed by experienced clinicians. Older adults with NC were recruited from the same hospital-based recruitment setting and were screened for the absence of neurological or psychiatric disorders.

### Inclusion and Exclusion Criteria

Inclusion criteria were being aged 60 to 70 years, completion of at least secondary-school education, ability to complete EEG recording, and ability to tolerate VR stimulation. For the AD and MCI groups, participants were required to have a clinical diagnosis established by experienced clinicians. For the NC group, participants were required to have no history of neurological or psychiatric disorders.

Exclusion criteria included severe visual impairment preventing VR participation, inability to complete EEG recordings, severe psychiatric disorders, major neurological diseases other than AD or MCI, and intolerance to VR stimulation. Participants were instructed to avoid medications or substances that could acutely affect EEG activity for at least 24 hours before the experiment whenever clinically appropriate.

### Participant Characteristics

A total of 60 older adults participated in this study, including 20 patients diagnosed with AD, 20 individuals with MCI, and 20 participants with NC. All participants were aged 60 to 70 years and had completed at least a secondary-school level of education. Sex distribution was balanced across groups, with 10 male and 10 female participants in each group.

Diagnoses of AD and MCI were established according to standard clinical diagnostic criteria by experienced clinicians. Although standardized cognitive screening scores such as the Mini-Mental State Examination (MMSE) were not available for all participants, the clinical recruitment process ensured that participants within each diagnostic group had comparable clinical presentations based on hospital assessment.

Prior to the formal experiment, all participants completed a 1-minute VR familiarization session using VR content different from that used during the main experiment. This procedure was performed to evaluate tolerance to VR stimulation and minimize adaptation effects. No participant reported dizziness, cybersickness, or other adverse reactions during the familiarization procedure. Visual function was screened prior to enrollment. All participants were able to complete the VR procedure without corrective eyewear and reported no visual difficulties during VR stimulation.

A participant flow diagram summarizing recruitment, eligibility screening, group allocation, VR-EEG recording, and final analysis is provided in Multimedia Appendix 1. Available demographic and clinical characteristics are summarized in Table 1.

**Table 1:** Demographic and clinical characteristics of the study participants.

Characteristic	AD^a^ group	MCI^b^ group	NC^c^ group
Sample size, n	20	20	20
Age (years), range	60-70	60-70	60-70
Education level	Secondary school or above	Secondary school or above	Secondary school or above
Sex (male/female), n	10/10	10/10	10/10
Recruitment source	Chuncheon Sacred Heart Hospital	Chuncheon Sacred Heart Hospital	Chuncheon Sacred Heart Hospital
Clinical comparability	Comparable clinical presentation within the AD group based on hospital assessment	Comparable clinical presentation within the MCI group based on hospital assessment	Control participants with NC
Visual function for VR^d^ participation	No severe visual impairment preventing VR participation	No severe visual impairment preventing VR participation	No severe visual impairment preventing VR participation
Corrective eyewear during VR	Not used during VR stimulation	Not used during VR stimulation	Not used during VR stimulation
VR familiarization	Completed 1-minute VR familiarization without adverse reaction	Completed 1-minute VR familiarization without adverse reaction	Completed 1-minute VR familiarization without adverse reaction
Final inclusion for EEG^e^ network analysis, n	20	20	20

^a^AD: Alzheimer disease.

^b^MCI: mild cognitive impairment.

^c^NC: normal cognition.

^d^VR: virtual reality.

^e^EEG: electroencephalography.

### Sample Size, Power, and Precision

Because this was an exploratory single-session, 3-group repeated-measures EEG study, no formal a priori power calculation was performed. The sample size was determined based on participant availability during the recruitment period and the feasibility of simultaneous VR-EEG recordings in older adults with cognitive impairment. The final sample included 20 participants in each diagnostic group, allowing balanced group-level exploratory comparisons of EEG-derived network metrics.

### Experimental Design and VR Stimulation

EEG data were collected using a 3-phase experimental paradigm consisting of a pre-VR resting state, a VR stimulation state, and a post-VR resting state.

VR stimulation was delivered using a PICO 4 head-mounted display (PICO Technology). Participants viewed an immersive 3D roller-coaster environment from a first-person perspective. The VR environment consisted of a passive first-person roller-coaster scenario that provided dynamic visual motion cues within an immersive 3D space. The paradigm was designed to provide visual and vestibular-like motion stimulation through optic flow, but participants were not required to perform motor responses, make decisions, or complete memory tasks. The VR stimulation lasted approximately 5 minutes. Participants remained seated comfortably in an upright position throughout the experiment.

Participants were monitored for discomfort, dizziness, and cybersickness during both the familiarization procedure and the main VR stimulation session. No participant discontinued the experiment because of VR-related discomfort. After the removal of the VR stimulus, a post-VR resting state was conducted to evaluate subsequent changes in brain activity. Each phase lasted approximately 5 to 6 minutes, and a 15-minute interval was provided between phases to minimize potential carryover effects.

The pre-VR, VR, and post-VR recordings were repeated within-session experimental states and did not represent longitudinal follow-up assessments.

### Measures and Covariates

The primary neurophysiological measures were EEG-derived graph-theoretical metrics, including GE, modularity, and nodal strength, calculated from PLV-based functional connectivity networks. Diagnostic group and experimental state were the main grouping variables. Standardized cognitive screening scores, such as MMSE, were not available for all participants and were therefore not included as covariates in the statistical analyses.

### EEG Data Acquisition

EEG signals were recorded using a HydroCel Geodesic Sensor Net (Electrical Geodesics) coupled with an EGI NetAmps 300 amplifier (Electrical Geodesics). A total of 64 electrodes were placed according to the International 10-20 system. Signals were sampled at 1000 Hz, and Cz was used as the reference electrode throughout acquisition.

Electrode impedance was maintained within clinical recording standards during data acquisition. Participants were instructed to remain seated, relaxed, and to minimize head, facial, and limb movements throughout recording. EEG data were collected during all 3 experimental states: pre-VR resting, VR stimulation, and post-VR resting. During the pre-VR and post-VR resting states, participants remained awake and visually fixated without active task performance. During VR stimulation, the headset was positioned carefully to avoid excessive pressure on the EEG electrodes and to minimize mechanical artifacts during simultaneous VR-EEG recording [31,32].

### EEG Preprocessing

EEG preprocessing was performed using EEGLAB. Raw EEG data were imported from EGI MFF files, and electrode locations were assigned using a 68-electrode channel-location file. Before independent component analysis (ICA), the data were high-pass filtered at 1 Hz. The data were then rereferenced using T7 and T8 as reference channels; if the specified reference channels were unavailable or failed validation, average referencing was used instead.

ICA-based artifact correction was performed using the extended runica algorithm in EEGLAB. To account for rank reduction after rereferencing, ICA decomposition was performed with principal component analysis (PCA) dimensionality reduction, using up to 64 principal components. Independent components were classified using ICLabel. Components classified as muscle, eye, heart, line-noise, or channel-noise artifacts with a probability of 0.90 or higher were automatically flagged and removed, whereas components classified as brain or other were not automatically rejected. Across the analyzed ICA-cleaned EEG recordings, the number of removed independent components was 7.68 (SD 7.32) per recording, with a median of 6 and a range of 0 to 32 components. After ICA-based artifact correction, the data were low-pass filtered at 70 Hz and saved as ICA-cleaned EEG datasets.

Following artifact correction, EEG signals were decomposed into multiple frequency bands, including δ (1-4 Hz), θ (4-8 Hz), α (8-13 Hz), β (13-32 Hz), γ (32-70 Hz), and a full-spectrum band (1-70 Hz) [33]. After preprocessing, artifact-free 1-second epochs were extracted for further analysis. For each participant and experimental phase, 30 clean epochs were selected and used for subsequent functional connectivity analysis.

### Functional Connectivity Analysis

Functional connectivity was quantified using the PLV, which measures the consistency of phase differences between 2 signals over time. For channels *i* and *j*, PLV was computed as:

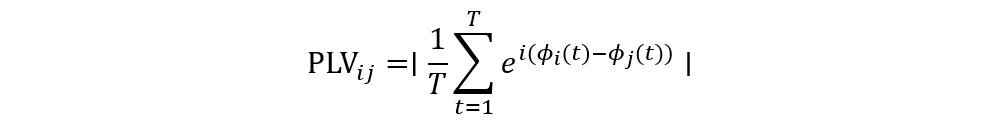



where *ϕ_i_*(*t*) and *ϕ_j_*(*t*) denote instantaneous phases obtained using the Hilbert transform. PLV values range from 0 (no phase synchronization) to 1 (perfect synchronization) [34].

### Network Construction and Thresholding

Functional brain networks were constructed based on PLV connectivity matrices derived from the preprocessed EEG signals. In the resulting network representation, each node corresponded to an EEG recording channel, and edges represented the PLV-based functional connectivity strength between pairs of channels.

For descriptive network visualization, a proportional thresholding approach was applied to the group-averaged PLV connectivity matrices. Specifically, only the top 5% of the strongest PLV connections were retained in the visualization graphs, while weaker connections were not displayed. This thresholding step was used to improve the interpretability of the network maps and to highlight the dominant spatial distribution of strong functional connections.

The 5% proportional threshold was selected as a literature-informed and visualization-oriented threshold. Previous EEG graph-theoretical studies have displayed only the strongest subset of functional connections to improve the interpretability of network topology and connection changes. For example, Liang et al [35] visualized only the upper 30% of mutual information values in a 19-channel EEG network to better illustrate network connection changes. In the present study, the number of EEG nodes and possible pairwise connections was substantially larger. Therefore, retaining higher proportions of connections, such as 20% or 10%, produced dense and visually cluttered graphs, with many overlapping edges that obscured the dominant spatial connectivity patterns. The 5% threshold provided the clearest visualization of the strongest PLV connections and regional network differences. Therefore, the top 5% of strongest PLV connections were retained only for network visualization and descriptive connectivity mapping. GE and modularity were calculated from weighted PLV matrices, whereas the top 5% proportional threshold was applied only to descriptive network visualizations.

### Graph-Theoretical Measures

#### Overview

Graph-theoretical metrics were calculated to characterize the topological properties of the constructed brain networks. In particular, GE was used to evaluate the efficiency of information integration across the entire network.

GE quantifies the capacity of a network to support parallel information transfer between distributed brain regions and is defined as:

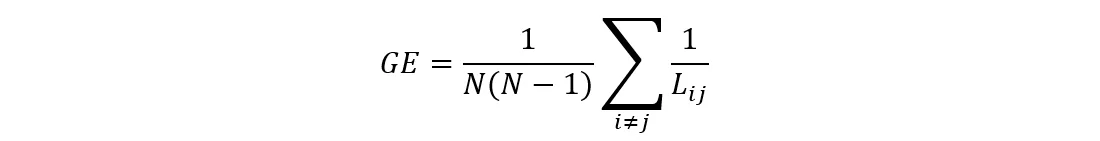



where 𝑁 represents the number of nodes in the network and *L*_i_*_j_* denotes the shortest path length between nodes *i* and 𝑗. A higher GE value indicates a more efficient network organization with stronger global integration and more effective communication between brain regions [36].

#### Modularity

Modularity measures the extent to which a network can be partitioned into densely connected modules with sparse intermodule connections. It is defined as:

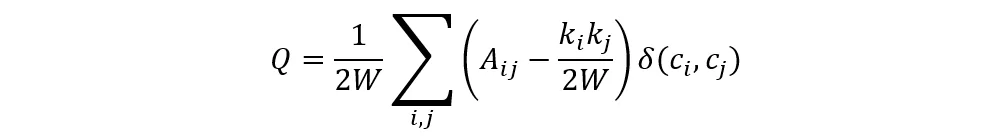



where *A*_i_*_j_* represents the edge weight between nodes *i* and *j*, *k*_i_ denotes node strength, *W* is the total edge weight, and δ(*c*_i_,*c_j_*) equals 1 when 2 nodes belong to the same module and 0 otherwise [37]. For modularity estimation, the Louvain algorithm was repeated 30 times, and the highest Q value was retained for each epoch.

#### Nodal Strength

Nodal strength was calculated as the sum of the weights of all edges connected to a given node, reflecting the overall level of connectivity of that node within the network [38].

### Visualization and Statistical Analysis

Brain network visualizations were generated to illustrate the spatial distribution of functional connectivity and to highlight long-range and interhemispheric connection patterns across experimental conditions. Connectivity graphs were projected onto scalp topography to provide an intuitive representation of network organization for each group and experimental state [35,39].

For statistical evaluation, graph-theoretical metrics were compared across diagnostic groups (AD, MCI, and NC) and experimental states (pre-VR resting, VR stimulation, and post-VR resting). Between-group differences within each experimental state were first assessed using 1-way ANOVA. When significant group effects were detected, post hoc pairwise comparisons using independent-sample 2-tailed *t* tests were performed to identify specific between-group differences. Within-group comparisons across experimental states were performed using paired-sample *t* tests. These statistical analyses were conducted to determine whether VR stimulation was associated with measurable modulation of brain network topology across the different cognitive states [40,41]. When reported, 95% CIs were calculated using the t distribution based on the corresponding group sample size.

Statistical significance was initially defined as *P*<.05. To control for multiple comparisons, *P* values were adjusted using the Benjamini-Hochberg false discovery rate (FDR) procedure [42]. FDR correction was applied separately within each graph-theoretical metric and comparison family. Specifically, between-group comparisons within the same experimental state and within-group comparisons across experimental states were treated as separate comparison families. Both raw *P* values and FDR-adjusted *P* values are reported where applicable.

### Missing Data

**Figure 1:**
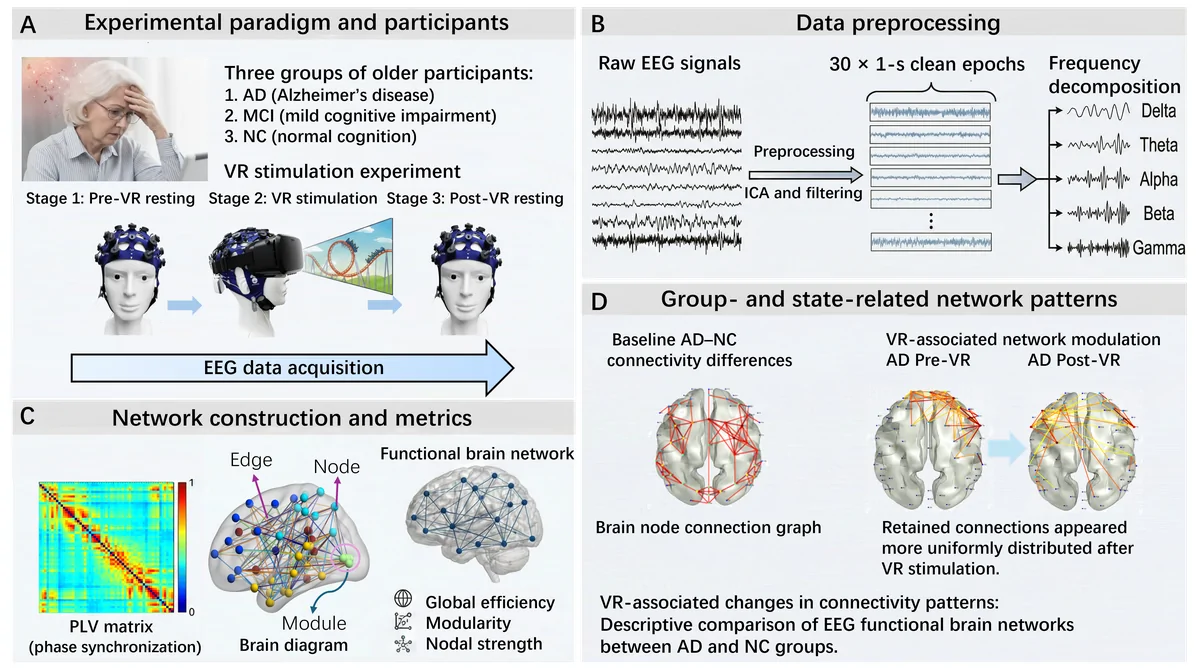
Virtual reality (VR)–based electroencephalography (EEG) network analysis workflow in AD. (A) Experimental paradigm and participants: EEG was recorded from AD, MCI, and NC groups during 3 stages: pre-VR resting, VR stimulation, and post-VR resting. (B) Data preprocessing: raw EEG signals were cleaned using preprocessing and independent component analysis (ICA) filtering, followed by frequency-band decomposition. (C) Network construction: functional brain networks were constructed using phase-locking value (PLV)–based connectivity, and graph metrics (global efficiency, modularity, and nodal strength) were calculated. (D) Group- and state-related network patterns: descriptive network differences between AD and NC were analyzed to illustrate VR-associated changes in connectivity distribution.

No participant-level data were missing in the final dataset. During EEG preprocessing, artifact-contaminated segments were removed according to the predefined quality-control procedures. For each participant and experimental condition, 30 artifact-free epochs were retained for subsequent analyses, ensuring an equal number of observations across groups and experimental states. The overall VR-EEG experimental and functional network analysis workflow is shown in Figure 1.

## Results

### Overview

Unless otherwise specified, inferential statistical conclusions were based on graph-theoretical metrics, including GE and modularity. PLV matrices, nodal strength distributions, and node connection graphs were used primarily as descriptive visualizations to illustrate spatial connectivity patterns and to support interpretation of the quantitative network results.

### PLV Matrix

The PLV connectivity matrices were used as descriptive visualizations to illustrate broadband functional connectivity patterns across diagnostic groups and experimental states. In the pre-VR resting state, the NC group visually showed stronger broadband coupling, whereas the AD group showed weaker and less distributed connectivity patterns, with the MCI group showing intermediate features.

During VR stimulation, the PLV matrices suggested a change in broadband connectivity organization, particularly in the NC and AD groups. In the NC group, broadband PLV patterns appeared less globally dense during VR stimulation, whereas the AD group showed relatively limited changes in overall coupling strength. In the post-VR resting state, visual differences among the 3 groups appeared less pronounced than those observed at baseline.

**Figure 2:**
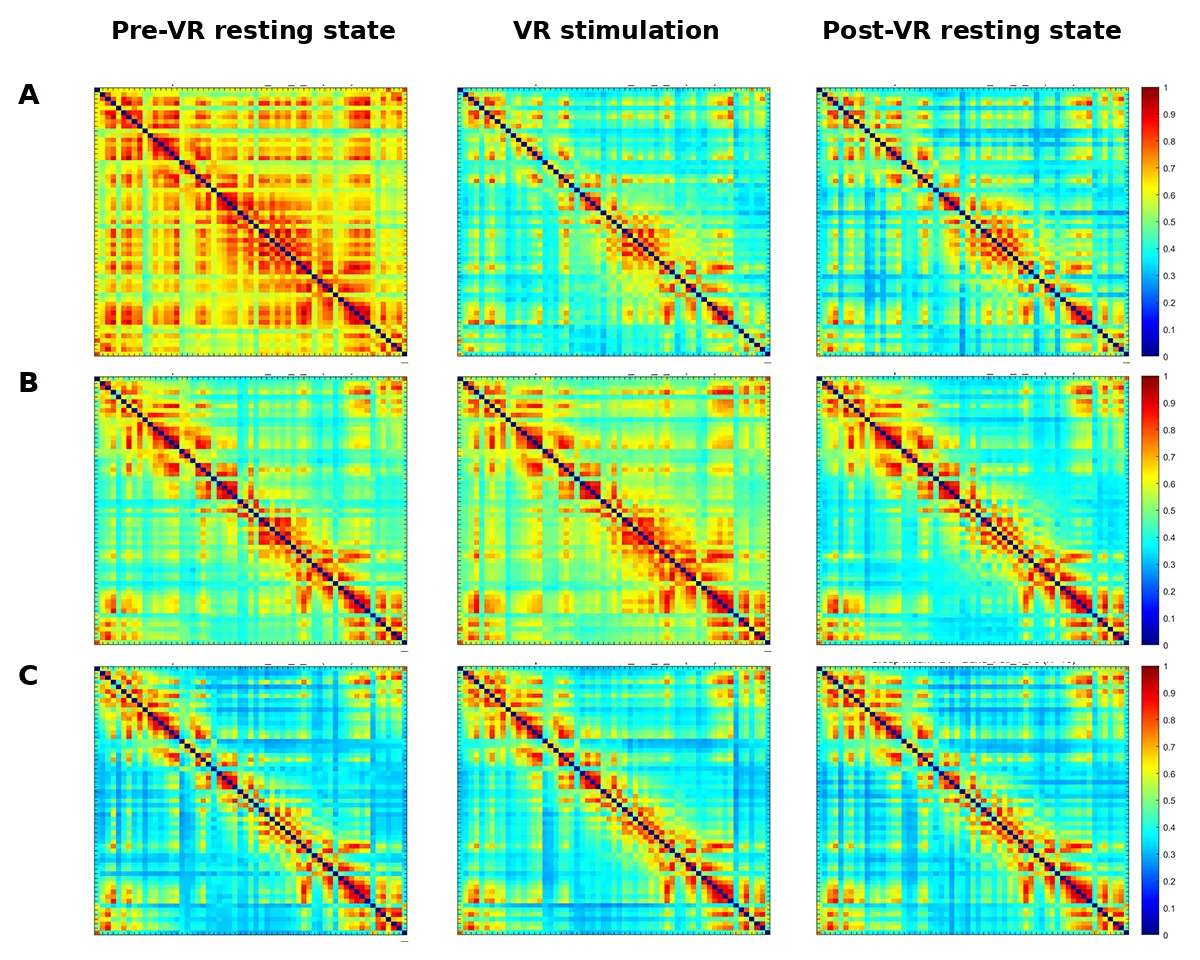
Phase-locking value (PLV)–based functional connectivity matrices across experimental stages. (A) PLV matrices of the normal cognition (NC) group. (B) PLV matrices of the Alzheimer disease (AD) group. (C) PLV matrices of the mild cognitive impairment (MCI) group. For each group, connectivity matrices are shown for 3 experimental stages: (1) pre–virtual reality (pre-VR) resting state, (2) during VR stimulation, and (3) post-VR resting state. The matrices represent functional connectivity in the full frequency band (1-70 Hz), where warmer colors indicate stronger phase synchronization between electroencephalography (EEG) channels.

Because these PLV matrices were intended primarily for visualization, formal statistical interpretation was based on subsequent graph-theoretical metrics, including GE and modularity. These descriptive PLV patterns provide spatial context for the quantitative network changes reported below. The broadband PLV connectivity matrices across diagnostic groups and experimental states are shown in Figure 2.

### GE Values

**Figure 3:**
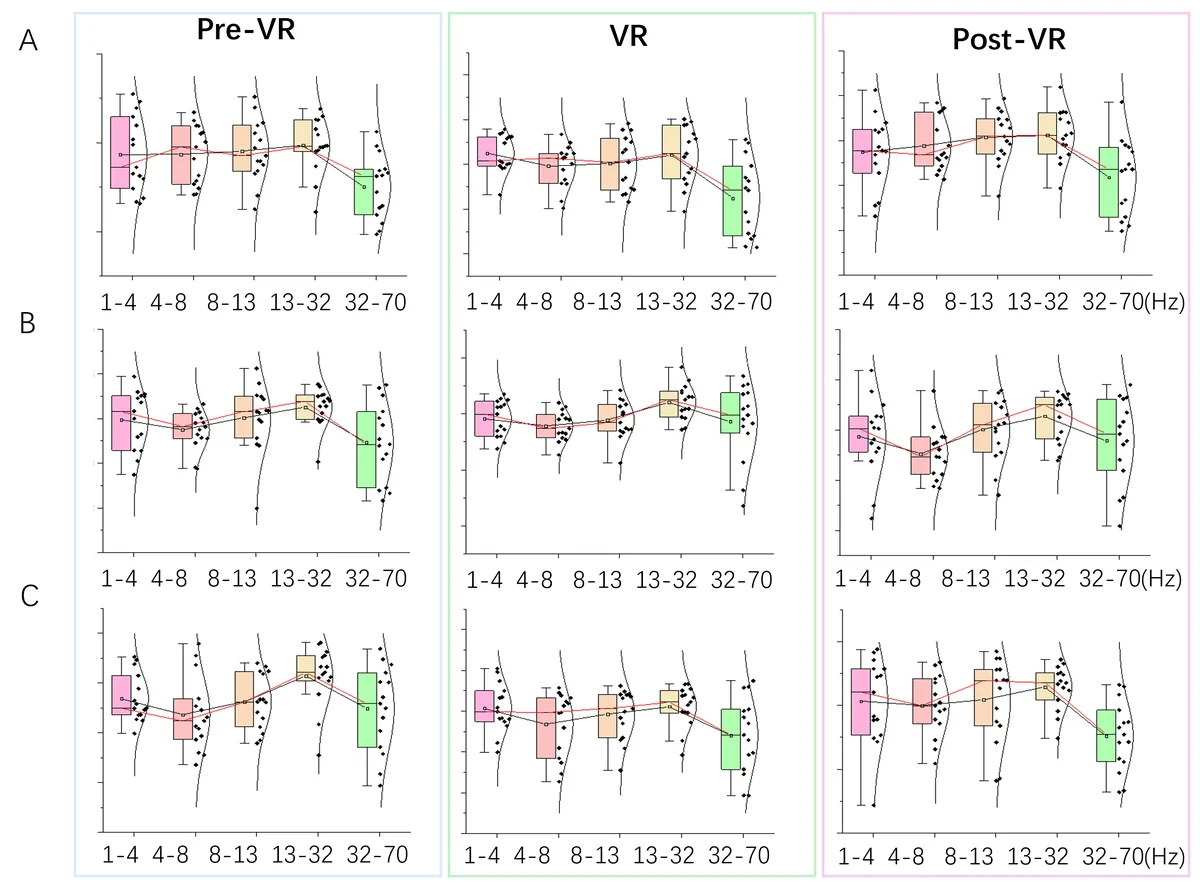
Global efficiency across frequency bands and experimental states. (A) Global efficiency values of the normal cognition (NC) group across frequency bands during the 3 experimental states: pre–virtual reality (pre-VR) resting, VR stimulation, and post-VR resting. (B) Global efficiency values of the mild cognitive impairment (MCI) group across the same frequency bands and experimental states. (C) Global efficiency values of the Alzheimer disease (AD) group across the 3 states.

Figure 3 illustrates GE values across frequency bands and experimental states in the NC, MCI, and AD groups. Descriptively, GE values tended to be higher in the 13 to 32 Hz range than in lower-frequency and higher-frequency bands across several groups and experimental states. This pattern suggests that beta-band activity may contribute to large-scale network integration in the present EEG dataset. However, because frequency-band comparisons were primarily descriptive, this observation should be interpreted cautiously and not as confirmatory evidence of a frequency-specific network mechanism.

In the broadband frequency range (1-70 Hz), between-group differences in GE were most evident during the pre-VR resting state. In the pre-VR condition, patients with AD exhibited higher GE than both NC participants (AD: 0.425, SD 0.036, 95% CI 0.408-0.442; NC: 0.337, SD 0.081, 95% CI 0.299-0.375; raw *P*=.009; FDR-adjusted *P*=.04) and patients with MCI (MCI: 0.343, SD 0.064, 95% CI 0.313-0.373; raw *P*=.003; FDR-adjusted *P*=.03). During VR stimulation, the difference in GE between the AD and NC groups was no longer significant (AD: 0.411, SD 0.033, 95% CI 0.396-0.426; NC: 0.412, SD 0.065, 95% CI 0.382-0.442). GE in the AD group was higher than that in the MCI group during VR stimulation in the uncorrected analysis (MCI: 0.355, SD 0.071, 95% CI 0.322-0.388; raw *P*=.04); however, this comparison did not remain significant after FDR correction (FDR-adjusted *P*=.12) and was therefore interpreted as an exploratory trend. In the post-VR state, GE values in the AD and NC groups converged, and no significant difference was observed between the 2 groups.

**Figure 4:**
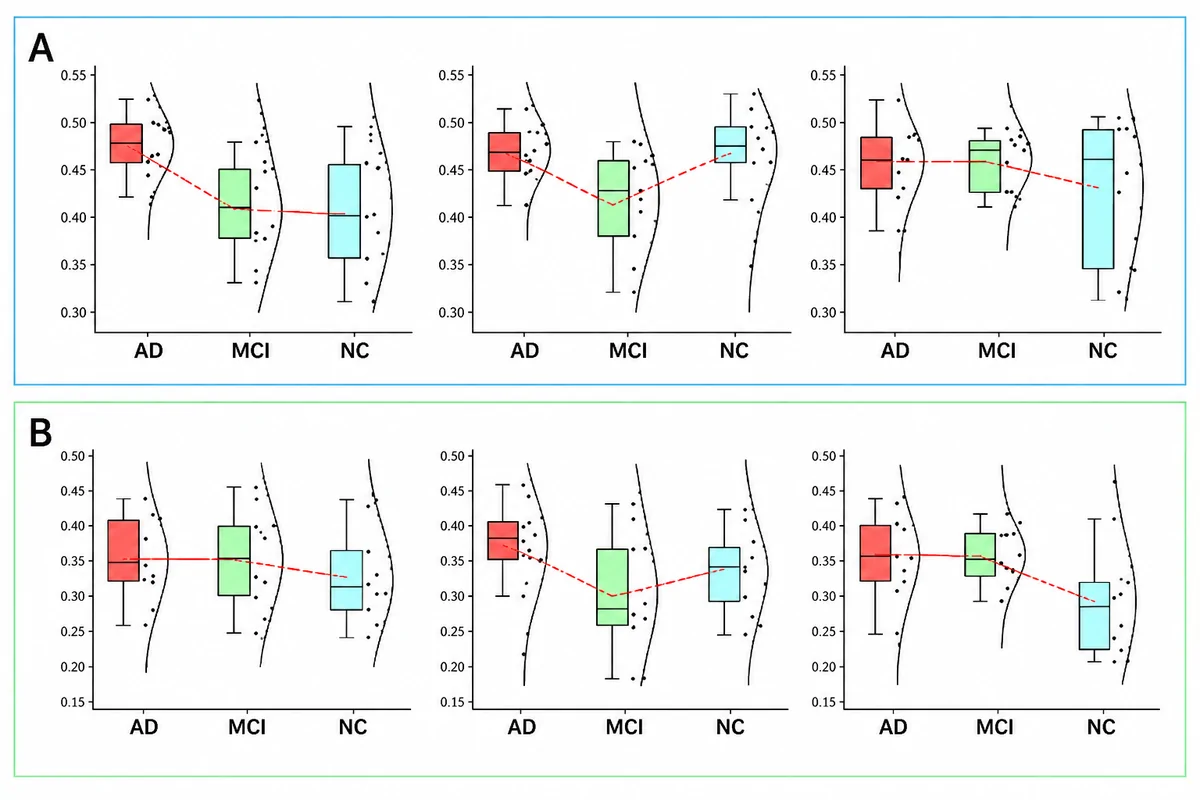
Group comparisons of global efficiency (GE) across experimental states. (A) GE comparisons among Alzheimer disease (AD), mild cognitive impairment (MCI), and normal cognition (NC) groups across the 3 experimental states (pre–virtual reality [pre-VR], VR stimulation, and post-VR) in the broadband frequency range (1-70 Hz). (B) Global efficiency comparisons among the 3 groups across the same experimental states in the gamma frequency band (32-70 Hz).

A comparable descriptive trend was observed in the high-frequency band (32-70 Hz); however, no between-group or within-group state comparison in this frequency band remained significant after FDR correction. Therefore, high-frequency GE findings were interpreted as exploratory patterns rather than confirmatory statistical effects. Across all experimental states, the MCI group generally exhibited intermediate GE values between the AD and NC groups, accompanied by moderate state-dependent variations. These results suggest that VR stimulation was associated with dynamic changes in network integration, with reduced AD-NC group differences in GE during and after VR stimulation. Each panel shows the distribution of GE values for the 3 diagnostic groups, illustrating group-level differences in network integration under different VR conditions in Figure 4.

These findings suggest distinct patterns of GE modulation across the clinical groups. At baseline, patients with AD exhibited elevated GE, suggesting altered or dysregulated network integration. During VR stimulation, GE in the AD group showed a decreasing trend; however, within-group GE changes in the AD group did not remain significant after FDR correction.

In contrast, participants in the NC group demonstrated a significant increase in GE during VR stimulation (pre-VR: 0.337, SD 0.081, 95% CI 0.299-0.375; VR: 0.412, SD 0.065, 95% CI 0.382-0.442; raw *P*=.007; FDR-adjusted *P*=.04). This increase suggests state-dependent upregulation of large-scale functional integration in response to sensory stimulation. The MCI group did not show an immediate increase in GE during the VR phase; instead, GE increased from pre-VR resting to the post-VR state after FDR correction (raw *P*=.009; FDR-adjusted *P*=.04), suggesting a delayed network response following stimulation.

**Figure 5:**
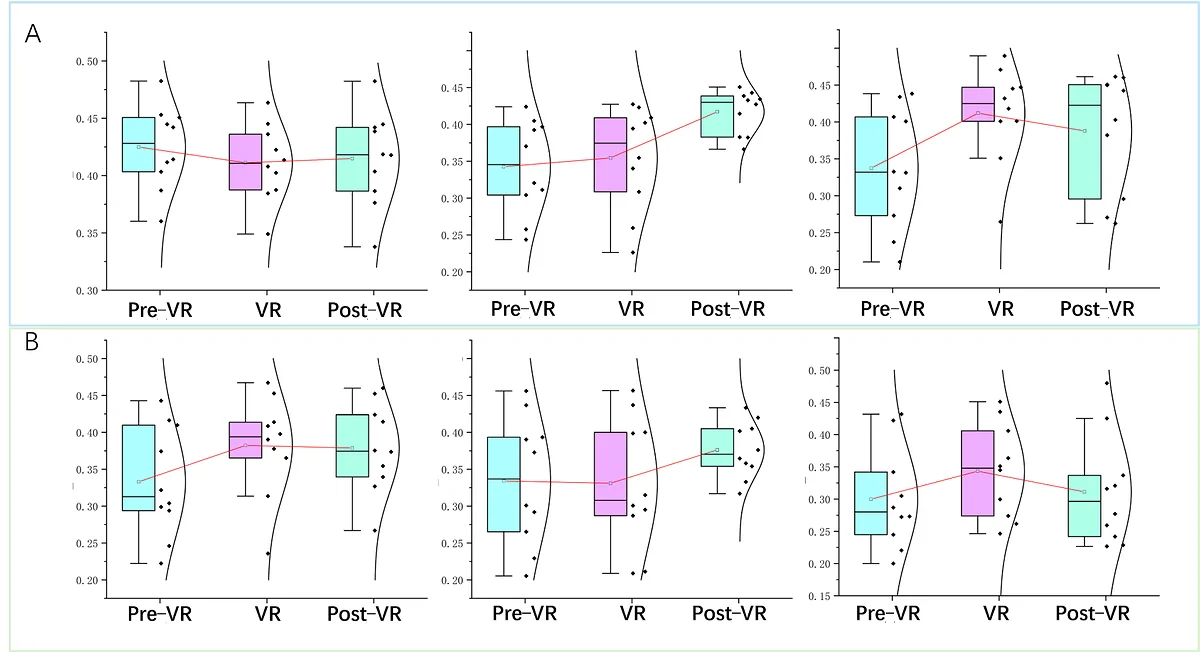
State-dependent changes in global efficiency (GE) within each clinical group. (A) GE across the 3 experimental states—pre–virtual reality (pre-VR) resting, VR stimulation, and post-VR resting—within each group (Alzheimer disease [AD], mild cognitive impairment [MCI], and normal cognition [NC]) in the broadband frequency range (1-70 Hz). (B) Global efficiency across the same experimental states within each group in the high-frequency band (32-70 Hz).

This temporal pattern suggests that network responsiveness in MCI may be partially preserved compared with AD, but that the dynamic regulation of global integration may occur more slowly. Taken together, these findings suggest a possible continuum of network dysfunction from NC to MCI to AD, characterized by rapid GE modulation in NC, delayed GE changes in MCI, and limited within-group GE modulation in AD. Each panel illustrates the distribution of GE values across experimental states, highlighting state-dependent modulation of network integration within the AD, MCI, and NC groups in Figure 5.

### Modularity

**Figure 6:**
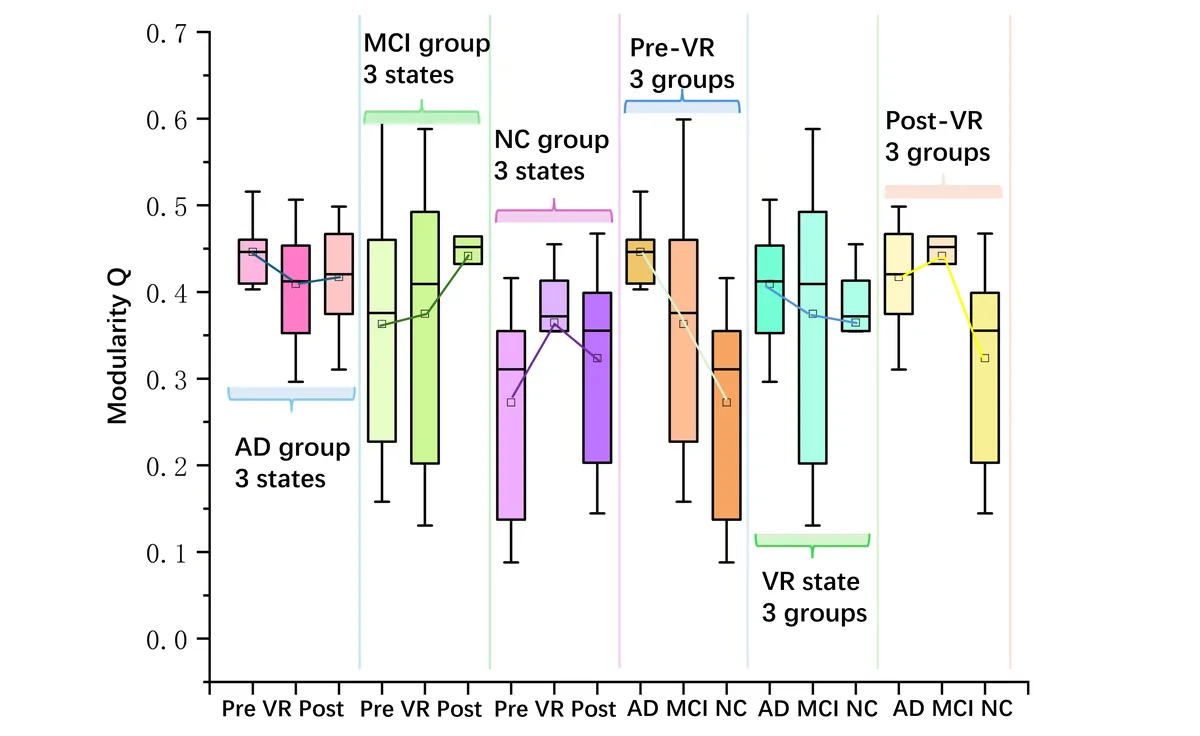
Comparison of modularity values across groups and experimental states. Modularity values are compared across the 3 experimental states (pre–virtual reality [pre-VR], VR, and post-VR) and the 3 clinical groups (Alzheimer disease [AD], mild cognitive impairment [MCI], and normal cognition [NC]). Boxplots illustrate state-dependent changes within each group and group differences within each experimental state. Only the baseline AD–NC modularity difference remained significant after false discovery rate (FDR) correction; the remaining group and state patterns are presented descriptively.

Modularity analysis revealed a robust baseline group difference and descriptive state-dependent patterns across clinical groups, partially paralleling the patterns observed for GE [43]. In the pre-VR resting state, patients with AD exhibited higher modularity than NC participants, and this difference remained significant after FDR correction (AD: 0.446, SD 0.036; NC: 0.273, SD 0.120; raw *P*=.001; FDR-adjusted *P*=.01), while the MCI group showed intermediate values (0.363, SD 0.146). During VR stimulation, modularity values converged across the 3 groups, and no significant between-group differences were observed after FDR correction. In the post-VR state, modularity values in the AD and MCI groups were comparable, and both were numerically higher than those of the NC group. The MCI-NC difference was significant in the uncorrected analysis but did not remain significant after FDR correction (raw *P*=.02; FDR-adjusted *P*=.07); therefore, this result was interpreted as an exploratory trend, as shown in Figure 6.

Within-group comparisons across experimental states showed descriptive temporal modulation patterns, but no within-group state comparison remained significant after FDR correction. In the AD group, modularity numerically decreased during VR stimulation, suggesting a possible reduction of the highly segregated network organization observed at baseline. The MCI group showed minimal numerical change during VR but exhibited higher modularity in the post-VR phase, suggesting a possible delayed network reconfiguration pattern. In contrast, participants in the NC group showed a numerical increase in modularity during VR stimulation followed by a reduction in the post-VR state. These within-group modularity patterns should therefore be interpreted as descriptive trends rather than confirmatory statistical effects.

### Nodal Strength

**Figure 7:**
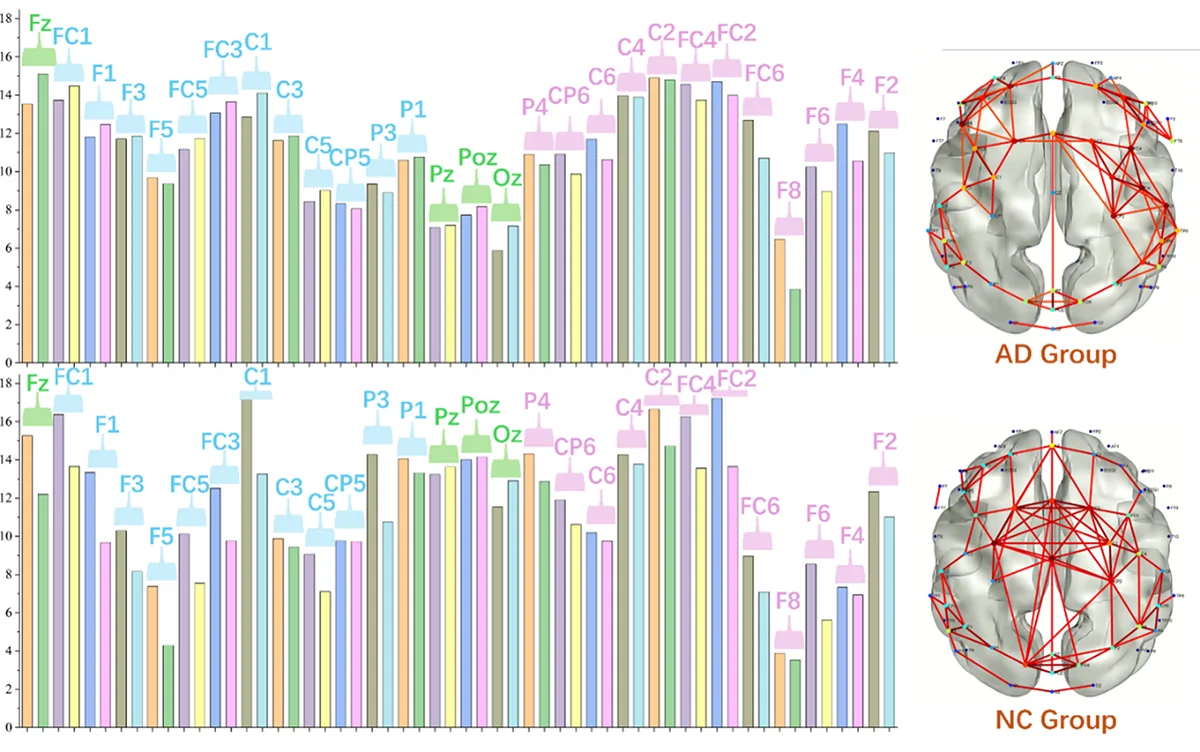
Regional node strength distribution and connectivity patterns in the Alzheimer disease (AD) and normal cognition (NC) groups. The upper panel shows the AD group, and the lower panel shows the NC group. For each group, node strength values are presented for 2 experimental states: pre–virtual reality (pre-VR) resting (left) and VR stimulation (right). Bars represent node strength for individual electroencephalography (EEG) channels. Node labels are color-coded by anatomical region: blue indicates left-hemisphere nodes, green indicates midline (central) nodes, and pink indicates right-hemisphere nodes. The brain network diagrams on the right illustrate the corresponding connectivity patterns for each group.

This study investigated alterations in EEG-based nodal connection strength during the pre-VR and VR conditions, illustrating VR-associated changes in functional brain network patterns in patients with AD. As shown in Figure 7, during the pre-VR resting state, the AD group exhibited pronounced right-lateralized connectivity, with higher nodal strength in the right hemisphere than in the left, whereas the NC group showed relatively balanced interhemispheric connectivity patterns.

During VR stimulation, the AD group demonstrated a bidirectional modulation pattern: most nodes in the left hemisphere showed increased connection strength, while several nodes in the right hemisphere displayed decreased strength. This “left-up and right-down” pattern suggested a shift in interhemispheric connectivity distribution during VR stimulation, with the AD connectivity pattern appearing more balanced than in the pre-VR resting state.

Analysis of key midline hub nodes (Pz, POz, Fz, and Oz) further revealed that the NC group exhibited variable changes with generally higher nodal strength, whereas the AD group showed consistent increases in nodal strength across these central regions during VR stimulation. As illustrated in Figure 7, these changes appeared to reduce visual group differences in these core integration nodes.

Taken together, these descriptive nodal strength patterns suggest that VR stimulation was associated with a shift in nodal connectivity distribution in AD, characterized by increased left-hemisphere and midline involvement and reduced apparent right-lateralized dominance. Because nodal strength maps were used primarily for descriptive visualization, these findings should be interpreted as spatial patterns of regional connectivity rather than evidence of therapeutic efficacy or functional improvement. These descriptive findings provide spatial context for the quantitative GE and modularity results, which indicated state-dependent changes in large-scale network organization.

### Node Connection Graph

Node connection graphs were used as descriptive visualizations of the top 5% strongest PLV connections and were not treated as independent inferential statistical tests or threshold-sensitivity analyses. Using preprocessed EEG data in the .set format and retaining the top 5% of strongest connections, the NC group showed stronger and more spatially distributed retained PLV connections than the MCI and AD groups, with relatively balanced interhemispheric organization. During VR stimulation, the retained connections in the NC group appeared more selective and spatially distributed across the network, suggesting a descriptive shift in the organization of strong PLV connections under sensory stimulation [44].

In contrast, the AD group exhibited marked hemispheric asymmetry at rest, with connectivity predominantly concentrated in the right hemisphere, particularly in central and centroparietal regions. This central and centroparietal concentration may reflect compensatory reliance on sensorimotor integration hubs and midline relay regions when distributed long-range coordination is reduced, consistent with more locally constrained coupling in disease. During VR stimulation, hemispheric asymmetry decreased, and the network adopted a more symmetric configuration, suggesting a state-associated shift toward a more bilateral distribution of retained connections and redistributed connectivity away from an overly lateralized resting-state pattern. The MCI group showed intermediate features between NC and AD but lacked a stable configuration across states, consistent with a transitional and heterogeneous network phenotype.

**Figure 8:**
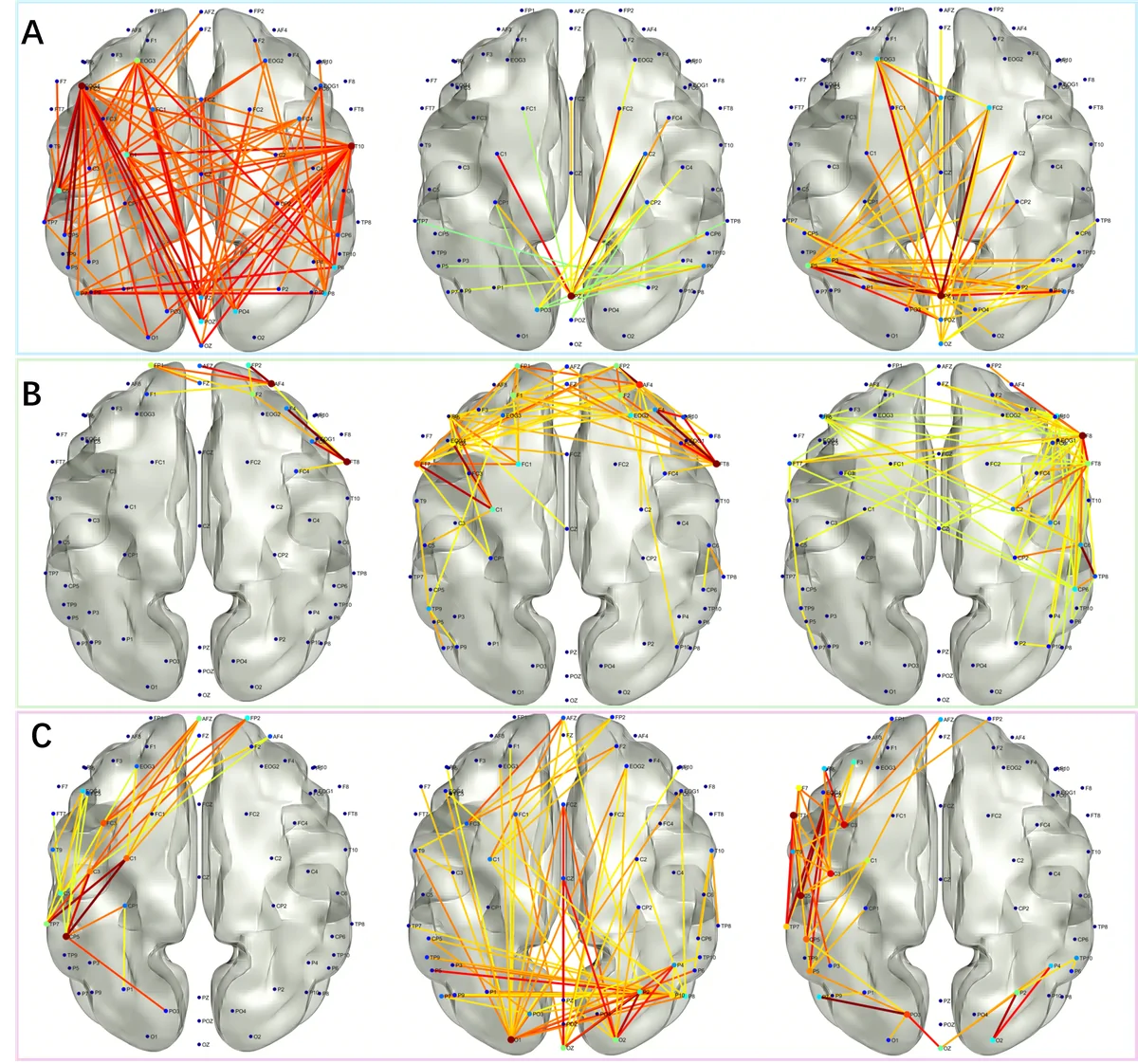
Brain network connectivity differences across groups and experimental states. The panels correspond to 3 frequency ranges: broadband (1-70 Hz), gamma band (32-70 Hz), and alpha band (8-13 Hz). For descriptive visualization, each network displays the top 5% of the strongest phase-locking value (PLV) connections. The maps were not treated as independent inferential evidence. (A) Connectivity difference maps obtained by subtracting the Alzheimer disease (AD) group from the normal cognition (NC) group across the 3 experimental states. (B) Connectivity difference maps obtained by subtracting the NC group from the AD group across the 3 experimental states. (C) Connectivity differences between the VR stimulation state and the resting state within the AD group.

Overall, VR stimulation was associated with a more selective and spatially balanced distribution of retained connections in NC networks. In AD, VR stimulation was associated with reduced apparent resting-state lateralization and a more bilateral distribution of retained connections. These connectivity reorganization patterns across groups and states are illustrated in Figure 8.

To further evaluate VR-related network changes in AD, differential connectivity maps were generated by subtracting the pre-VR network from the VR-state network within the AD group across frequency bands. In the broadband range (1-70 Hz) and in the mid-frequency band (8-13 Hz), VR-related connectivity increases were mainly localized to the left hemisphere, with prominent involvement of central and centroparietal regions. This pattern contrasts with the right-hemisphere dominance observed at rest and suggests a state-associated shift toward more bilateral hemispheric distribution.

In the high-frequency band (32-70 Hz), descriptive maps showed a different spatial distribution of retained long-range connections during VR stimulation. Because high-frequency GE findings did not remain significant after FDR correction, this pattern should be interpreted as visualization-based spatial context rather than confirmatory evidence of enhanced high-frequency communication.

Taken together, the node connection graphs descriptively suggested that VR stimulation was associated with a shift in AD connectivity patterns from a more lateralized resting-state configuration toward a more bilateral distribution. These visualization-based observations should be interpreted together with the quantitative GE and modularity findings rather than as independent statistical evidence. Overall, the connectivity maps provide spatial support for the interpretation that VR stimulation was associated with short-term changes in large-scale functional network organization.

## Discussion

Across the 3 within-session experimental states, this exploratory single-session, 3-group repeated-measures EEG study identified FDR-corrected baseline group differences in broadband GE and modularity, together with FDR-corrected within-group changes in GE in the NC and MCI groups. Descriptive connectivity maps also suggested state-associated changes in hemispheric distribution and high-frequency connectivity; however, the corresponding high-frequency GE findings did not survive FDR correction and were therefore treated as exploratory. Collectively, these results support the feasibility of using immersive VR as a controlled challenge for examining acute EEG network responsiveness, while not establishing a therapeutic effect.

At baseline, the AD group showed elevated GE and modularity relative to the NC group, indicating altered organization of network integration and segregation. Prior EEG network studies and reviews have similarly reported disease-related changes in synchronization, coherence, efficiency, modular organization, and hub connectivity in AD and MCI [20,45-47]. During VR exposure, the apparent separation between groups was reduced for some measures; however, the absence of a statistically significant difference should not be interpreted as normalization, equivalence, or functional recovery.

Although higher GE is commonly interpreted as greater network integration, elevated GE in AD does not necessarily represent beneficial or more efficient neural processing. In a disrupted neurodegenerative network, greater apparent integration may reflect altered synchronization, compensatory recruitment, reduced modular constraints, or pathological overintegration [20,36,45-47]. Accordingly, the elevated baseline GE observed in AD is interpreted as altered network organization rather than enhanced functional performance.

Descriptive maps suggested that the spatial distribution of strong connections became more bilateral during VR exposure than during the pre-VR resting state. Previous work has linked AD and MCI to hemispheric imbalance and disrupted interhemispheric coordination [40,41,45]. Because the present maps were descriptive and dependent on the selected display threshold, they provide spatial context for the quantitative findings rather than confirmatory evidence of hemispheric rebalancing.

Descriptive high-frequency maps suggested state-associated redistribution of strong connections; however, high-frequency GE findings did not remain significant after FDR correction. Task-based and connectivity-focused EEG literature supports the possibility that cognitive or sensory challenges may reveal neurophysiological features not evident during resting-state recording [46-49]. Nevertheless, the present high-frequency observations should be considered hypothesis-generating rather than evidence of a confirmed frequency-specific mechanism.

The NC and MCI groups showed FDR-corrected within-group GE changes at different phases of the experiment, whereas no within-group change in AD remained significant after correction. These findings may indicate group-related differences in acute network responsiveness, consistent with the heterogeneity described across NC, MCI, and AD [21,22,45-47,49]. However, without a direct group-by-state interaction test, the observed patterns should not be interpreted as a confirmed progression continuum or as proof that one group responded more strongly than another.

A contribution of the present study is the application of a common graph-theoretical framework across 3 clinical groups and 3 within-session experimental states. Prior EEG studies have demonstrated the relevance of spectral and connectivity measures for identifying cognitive decline in MCI and AD [46-52], whereas VR studies in older adults have primarily emphasized cognition, usability, training outcomes, or regional activation [10,11,23-25]. The present work extends this literature by examining acute, large-scale EEG network organization during and after immersive VR exposure.

Because concurrent cognitive, behavioral, functional, and clinical outcomes were not collected, the observed EEG changes cannot be directly linked to cognitive improvement, symptom severity, daily functioning, or therapeutic benefit. The practical significance of the study therefore lies in demonstrating the feasibility of combining immersive VR with EEG graph-theoretical analysis as an experimental challenge paradigm for characterizing short-term neurophysiological responsiveness. Future controlled and longitudinal studies should test whether these network measures are associated with standardized cognitive and everyday-functioning outcomes [10,11,23-25,49].

Methodological factors may also contribute to the observed connectivity patterns. PLV-based connectivity can be influenced by volume conduction, common-reference effects, signal leakage, filtering, and preprocessing decisions [34]. Graph-theoretical findings may further depend on edge weighting, node and edge definitions, network density, and proportional threshold selection [35,53]. The top 5% threshold was used only for descriptive visualization; because formal threshold-sensitivity analysis was not performed, the spatial maps should not be interpreted as threshold-independent biomarkers. Replication using multiple network densities, alternative connectivity estimators, and independent datasets is required.

Several limitations should be considered. First, the modest sample and convenience recruitment may limit generalizability, and complete individual-level demographic and standardized cognitive data were unavailable for all participants. Second, the study used a single-session repeated-measures design without a separate non-VR comparison condition; therefore, VR-specific effects cannot be separated from repeated recording, visual stimulation, novelty, arousal, fatigue, or other time-related influences. Third, connectivity was estimated using PLV, which can be affected by volume conduction, reference selection, and signal leakage [34]. Fourth, the top 5% proportional threshold was used only for descriptive visualization, and formal threshold-sensitivity analysis was not performed [53]. Fifth, a standardized cybersickness questionnaire was not administered, so mild discomfort or vestibular symptoms could not be quantified. Finally, no concurrent cognitive, behavioral, functional, or clinical outcomes or longitudinal follow-up were available. These limitations support interpreting the findings as exploratory and hypothesis-generating.

In conclusion, this exploratory single-session, 3-group repeated-measures EEG study identified short-term, state-associated changes in PLV-derived EEG functional network organization across older adults with AD, MCI, and NC. The findings support the feasibility of combining immersive VR with EEG graph-theoretical analysis to characterize acute network responsiveness. However, the descriptive connectivity maps were not treated as confirmatory evidence, and the lack of a non-VR comparison condition and concurrent cognitive or clinical outcomes precludes conclusions regarding causality, functional benefit, or therapeutic efficacy. Larger controlled and longitudinal studies are required to determine the reproducibility, functional significance, and potential clinical value of these network responses.

## Appendix

Multimedia Appendix 1 Participant flow diagram summarizing recruitment, eligibility screening, group allocation, and final analysis.

## Abbreviations

AD: Alzheimer disease
EEG: electroencephalography
FDR: false discovery rate
GE: global efficiency
ICA: independent component analysis
IRB: Institutional Review Board
MCI: mild cognitive impairment
MMSE: Mini-Mental State Examination
NC: normal cognition
PCA: principal component analysis
PLV: phase-locking value
VR: virtual reality

## Data Availability

Electroencephalography data and derived network metrics, including global efficiency, modularity, and nodal strength, are available upon reasonable request and with approval from the Institutional Review Board (IRB) of Kwangwoon University, Seoul, Republic of Korea, under IRB number 7001546-202300614-HR(SB)-005-01.
